# The Moderating Effect of Managerial Roles on Job Stress and Satisfaction by Employees’ Employment Type

**DOI:** 10.3390/ijerph17218259

**Published:** 2020-11-09

**Authors:** Kwan-Woo Kim, Yoon-Ho Cho

**Affiliations:** 1 Occupational Safety and Health Training Institute, Korea Occupational Safety & Health Agency (KOSHA), 400 Jongga-ro, Jung-gu, Ulsan 44429, Korea; 2 Occupational Safety and Health Research Institute, Korea Occupational Safety & Health Agency (KOSHA), 400 Jongga-ro, Jung-gu, Ulsan 44429, Korea

**Keywords:** employment type, job satisfaction, job stress, manager role, employees

## Abstract

The roles of managers affect job stress and satisfaction. As irregular employees increase globally, more research is needed on the effects of managerial roles. This study analyzed job stress (subfactors: job autonomy and demand), job satisfaction, and managerial roles by employment type. Data comprised 33,420 cases from the fifth Korean Working Condition Survey. Regular employees had higher job autonomy and satisfaction fewer lower demands than irregular employees. For both, job autonomy positively and job demand negatively affected job satisfaction; the interaction of job autonomy and managerial roles negatively affected the relationship between job autonomy and satisfaction. In the relationship between job demand and satisfaction, the interaction of job demand and managerial roles had positive and negative effects for regular and irregular employees, respectively. The moderating effect of the interaction between job stress and managerial roles differed by employment type. Thus, managerial roles should differ by employment type. Guaranteed autonomy and minimal managerial intervention positively affect job satisfaction regardless of employment type. Appropriate managerial intervention relieves job stress and increases satisfaction for regular employees; managerial intervention negatively impacts irregular employees’ satisfaction. Irregular employees should be provided with clear job expectations from the start, with minimal managerial intervention.

## 1. Introduction

Stress is becoming increasingly common in the everyday lives of modern individuals. It is estimated that approximately 50–70% of current diseases are correlated with stress [1]. Furthermore, some research has reported that 50–60% of lost workdays are because of job stress [2]. Stress also greatly influences the relationship between employees and their professional organization.

Many researchers concur that various situations within a company, such as working conditions and leadership, cause job stress [3,4]. Job stressors are determined by environmental factors, such as available alternative resources and workload [5,6]. Job stress was found to be the most important factor affecting turnover intention for Korean nurses. In a study of Australian alcohol and pharmaceutical employees, one in five reported above average stress levels and expressed turnover intentions [7,8]. Moreover, company costs increase owing to new hiring and training caused by frequent turnover [9]. Job stress not only affects work performance, job satisfaction, and organizational commitment; it also affects behavior, psyche, personal problems, and issues within an organization [10]. In addition, job stress has a negative effect on job satisfaction [11,12], while job satisfaction moderates the relationship between creativity and turnover intention, and decreases turnover intention [13,14,15,16]. From an organizational perspective, job satisfaction moderates the relationships among turnover intention and person-organization fit, employers’ perception of an organization’s external reputation, and organization identification [17,18,19]. 

Regular employees are full-time employees who have signed on with a company for an indefinite period of work. Irregular employees are all other employees, such as those who have a labor contract with a dispatching company, a finite labor contract with a fixed period, or a temporary job [20].

One characteristic of modern Korean society is an increase in irregular employees. As of August 2019, irregular employees accounted for 36.4% of all employees in Korea [21]. Some reasons for this phenomenon are employment costs and labor flexibility [22,23]. The most important reason for hiring irregular employees has been reported to be the reduction of labor costs [24]. Companies in Korea place a high level of importance on labor flexibility because regular employees, who receive relatively high levels of employment protection from government policies, corporate practices, social customs, and labor unions, are often difficult to dismiss, resulting in high labor costs [24].

The number of irregular employees is increasing globally, and their low wages, unstable employment status, and poor working conditions often lead to job stress [21]. As job stress affects society as a whole [25,26,27], better job stress management for irregular employees, who have a relatively weak social status, is urgently required. There are many factors that affect employees’ job stress and satisfaction, including the role of managers who communicate directly with employees. Managerial roles are becoming an important factor in responding to human resource management for organization management [28]. Managers support employees expend additional effort to achieve performance beyond expectations and improve the effectiveness of the organization [29]. Managers have important roles in coordinating and integrating the efforts of individual employees, by taking into account those employees’ characteristics [30].

Furthermore, job stress and satisfaction greatly influence not only individuals, but also families and companies; thus, many studies have been conducted to explore factors related to relieving employee job stress. Nevertheless, as the proportion of irregular employees gradually increases, more varied studies on irregular employees’ job stress and satisfaction are needed. Additionally, although there have been many studies on the roles of managers, such as on the relationships among leadership and job attitudes and service quality, organizational performance, and organizational commitment and group cohesion, as well as analyses of the leadership effect [31,32,33], most used samples from specific populations. Thus, it would be unreasonable to generalize, based upon the results of research on specific groups, regarding more widespread phenomena in society. Moreover, there has been little research on the relationship between job stress and satisfaction or, more specifically, the relationship between job stress and satisfaction according to managerial roles, for both regular and irregular employees. 

Therefore, this study aims to analyze the effects of managerial roles on job stress and satisfaction, as well as the characteristics of job stress and satisfaction by employment type, by utilizing data from a survey targeting all regions of a country, rather than a specific group. These results could then be used to inform company policy decisions regarding employee job stress and satisfaction.

## 2. Literature Review and Hypothesis Development

### 2.1. Job Stress and Job Satisfaction

Job stress can be defined as a situation in which employees’ characteristics interact with their jobs, resulting in psychological or physiological changes that negatively affect their functional abilities [10]. Job stress occurs when employees are in a work environment that does not match their motivations or abilities [34]. When employees cannot cope well with job stress, it can lead to issues that negatively impact both physical and psychological health, such as depression, anxiety, tension, headaches, alcohol use, and smoking [35]. Furthermore, job stress can lead to decreased job performance and low job satisfaction [36].

Job satisfaction can be defined as a state of mind determined by the extent to which people like or dislike their job or can meet their job-related needs [37,38]. Job satisfaction has been shown to have a positive effect on employee performance by influencing the formation of a favorable attitude toward an organization, ultimately leading to decreases in turnover and absenteeism rates, and improved cohesion among organization members [39].

Currently, the proportion of irregular employees is increasing; irregular employees experience relatively unstable employment status, compared with regular employees. Job insecurity increases anxiety and stress, and negatively affects job satisfaction [40,41]. One of the main reasons why organizations hire irregular employees is employment flexibility. Irregular employees with short working periods will inevitably differ from regular employees in job responsibilities, diversity, autonomy, and importance [22,23]. Job responsibilities, diversity, autonomy, and importance can cause differences in job stress and satisfaction between irregular and regular employees. Furthermore, job insecurity, work overload, role conflicts, and the lack of job autonomy have been found to greatly influence physical and mental stress among irregular employees [42].

Considering job stress to be a result of the interaction between environmental factors and individual reactions, demographic variables such as gender, age, and academic background were included in the scope of previous research to broaden our understanding of job stress [43]. The previous research confirmed that demographic characteristics affect job stress and satisfaction. In studies of specific groups, such as public corporation employees, teachers, vocational rehabilitation employees, aircraft crew members, special security guards, and female general hospital office employees, job stress and satisfaction were found to have a negative relationship [44,45,46,47,48,49]. However, other studies indicated that job stress does not consistently negatively affect job satisfaction [50,51]. With an appropriate level of job stress, a positive effect was found, but job stress that was too low or too high created a negative effect [52].

Therefore, the following hypotheses were established:

**Hypothesis 1 (H1)**. *There will be differences in job stress and satisfaction between regular and irregular employees.*

**Hypothesis 2 (H2)**. *There will be differences in job stress and satisfaction between regular and irregular employees according to demographic characteristics.*

**Hypothesis 3 (H3)**. *Job stress will affect job satisfaction in both regular and irregular employees.*

### 2.2. Manager Roles

Managerial roles are expressed in a manager’s leadership style, in terms of how it contributes to achieving organizational goals by influencing employees and motivating them to work hard. In other words, managerial roles can be considered a form of leadership [29]. Managerial roles can also be divided into transformative leadership, which gives employees maximum autonomy to make judgements and work on their own, in order to reach organizational goals, and transactional leadership, in which managers use compensation and punishment for each employee to enhance their job performance [29,53]. Transformation and transactional leadership styles have very different characteristics; however, as they are not mutually exclusive concepts, in order for managerial roles to be effective, it has been recommended that both leadership styles be appropriately combined according to the relevant circumstances [54,55,56]. Since leadership itself affects employees, employee feelings vary depending on leadership style. When managers make decisions with sincerity and based upon objective analyses, employees naturally trust them. Trust in managers has been shown to have a positive impact on employee job satisfaction [57]. Previous research found that the roles of hotel kitchen and restaurant industry managers positively affected employee job satisfaction [29,58]. However, while some studies have shown that leadership has a positive effect on job satisfaction, others have shown the opposite [59,60].

Previous research results suggested a relationship between managerial roles and employee job satisfaction. Therefore, the following hypothesis was established:

**Hypothesis 4 (H4)**. *Managerial roles will play a moderating role in the relationship between job stress and satisfaction in both regular and irregular employees.*

**Figure 1:**
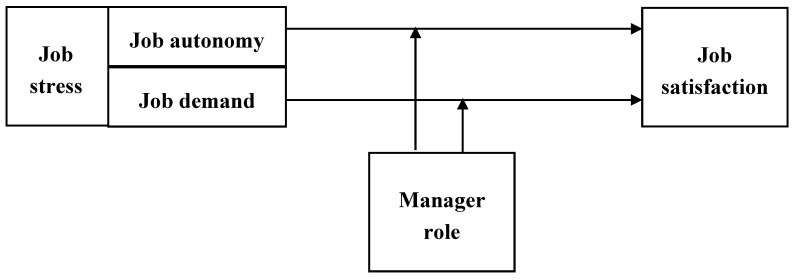
Research framework.

The moderating effect model of managerial roles in the relationship between job stress and satisfaction by employment type is shown in Figure 1.

## 3. Methods

### 3.1. Data Collection

Data were collected from the fifth Korean Working Condition Survey (KWCS), 2017. The KWCS is conducted every three years, and is based on the European Working Condition Survey (EWCS) implemented by Eurofound. The KWCS surveyed people 15 years and older with jobs; the survey was conducted by a professional surveyor through home-visits and one-on-one interviews. The KWCS has been used as Korea’s official national statistical data.

For this study, out of 52,205 cases in the KWCS, 33,420 in which the respondents were confirmed to be employees were analyzed. Out of these, regular and irregular employees accounted for 27,782 (83.1%) and 5638 (16.9%) cases, respectively.

### 3.2. Measurements

To measure job stress, we used related questions from the KWCS with reference to the Korean occupational stress scale [61]. The Korean occupational stress measurement scale includes job demand and autonomy as subfactors that measure job stress [62]. Physical working environment, job insecurity, and inadequate compensation are variables that can influence job stress, in addition to job demand and autonomy. Job demand and autonomy are factors related to job stress that could be extracted from the KWCS data. Therefore, job demand and autonomy were used as subfactors of job stress in this study. Job demand refers to the degree of burden employees experience on the job, and generally includes a factor such as time-pressure. Job autonomy refers to the level of decision-making authority and discretion that employees have in their jobs [62]. Therefore, the job demand item consisted of time-pressure, while the job autonomy items consisted of workload-control and decision-making from the KWCS. Job autonomy used the average value of workload-control and decision-making. For job demand and autonomy, a five-point Likert scale was used (1 = never and 5 = always).

To measure job satisfaction, we used five questions from the KWCS with reference to the research to measure Korean job satisfaction [63]. Thus, the items on job satisfaction consisted of wages, prospects, recognition, human relations, and motivation. Job satisfaction was measured using the average value of these five variables.

In the present study, “managerial roles” refers to the leadership of an immediate boss. Questions that measured leadership based on Korean sentiment were used [64]. Therefore, regarding managerial roles, six questions from the KWCS were used with reference to a previous study [64]. The items on managerial roles consisted of respect, compliment, cooperation, support, feedback, and encouragement. Managerial roles were measured using the average value of these six variables. For job satisfaction and managerial roles, a five-point Likert scale was used (1 = strongly disagree and 5 = strongly agree). Gender, age, academic background, and company size were used as demographic variables.

In order to verify the reliability of each variable, Cronbach’s α was used as a coefficient to measure the internal consistency, and the validity was verified through exploratory factor analysis. Principal component analysis applying varimax rotation was used for factor extraction. Nunnally suggested more than 0.70 as a stable reliability, but generally, more than 0.60 is accepted as an analytical level [65,66]. As shown in Table 1, Cronbach’s α in this study ranged from 0.740 to 0.880. Reliability analysis is a method to verify whether multiple items are consistent. Therefore, since the subfactor of job demand is one item (time-pressure), a reliability analysis for job demand was excluded.

### 3.3. Analyses

First, to examine H1 and H2, this study analyzed whether there were differences in job stress and satisfaction according to employment type, and whether there was an effect of demographic variables on the same by performing t-tests. Second, to examine H3, the study analyzed the effect of job stress on job satisfaction by employment type through multiple regression analyses. Third, to examine H4, hierarchical regression analyses were conducted to ascertain whether managerial roles moderated the relationship between job stress and satisfaction by employment type. SPSS 18.0 (IBM, Armonk, NY, USA) was used for statistical analyses. Significance was set at *p* < 0.05.

## 4. Results

### 4.1. Characteristics of Job Stress and Satisfaction by Employment Type

The differences in job stress and satisfaction between regular and irregular employees are presented in Table 2. There were significant differences between regular and irregular employees in job autonomy and job demand, which are subfactors of job stress, thereby supporting H1. The job autonomy of regular employees was higher than that of irregular employees. Both workload-control and decision-making, which are subfactors of job autonomy, were higher in regular employees than irregular employees. Moreover, the time-pressure of job demands was higher in irregular employees than regular employees. Additionally, there were significant differences between the job satisfaction among regular and irregular employees, with that of the former being higher than that of the latter, also supporting H1.

The differences in job stress and satisfaction between regular and irregular employees by gender, age, academic background, and company size are presented in Table 3. The job autonomy of regular employees was higher than that of irregular employees across gender, age, academic background, and company size, thus supporting H2. Job autonomy was higher as academic background increased in both regular and irregular employees. For regular employees, but not irregular employees, it was found that the larger the company size, the higher the job autonomy. The job demand of irregular employees was higher than that of regular employees across gender, age, academic background, and company size, also supporting H2. For regular employees, the higher the academic background and the larger company size, the lower the job demand, which was not the case for irregular employees. The job satisfaction of regular employees was higher than that of irregular employees across gender, age, academic background, and company size, further supporting H2. For regular employees, the higher the academic background and the larger company size, the higher the job satisfaction; however, this was not the case for irregular employees.

### 4.2. Effects of Individual Variables of Job Stress on Job Satisfaction by Employment Type

This study analyzed whether job stress affected job satisfaction based on employment type. That is, analyses were conducted on whether workload-control, decision-making, and time-pressure, the subfactors of job autonomy and job demand, affected job satisfaction. The job stress of regular and irregular employees had a significant effect on job satisfaction, thereby supporting H3. Table 4 shows the effects of job stress on job satisfaction between regular and irregular employees. Among both groups, workload-control and decision-making had a positive effect on job satisfaction. However, time-pressure had a negative effect on job satisfaction in both regular and irregular employees. The magnitude of the influence on job stress was in the order of time-pressure, workload-control, and decision-making for both regular and irregular employees.

### 4.3. Moderating Effects of Managerial Roles on Job Satisfaction by Employment Type

Table 5 shows the results concerning the question of whether the effects of regular employee job stress on job satisfaction were moderated by managers. In Model 1, only control variables were included. In Model 2, independent variables (job autonomy and job demand) were added. In Model 3, a moderating variable (manager roles) was further added. The interactions between job autonomy and managerial roles, and between job demand and managerial roles, were added in Model 4. The fit of all models was statistically significant. The explanatory power of Model 2 increased by 9.7% compared to Model 1: Job autonomy had a positive effect on job satisfaction. However, job demand had a negative effect on job satisfaction. The explanatory power of Model 3 increased by 15.5% compared to Model 2: Managerial roles had a positive effect on job satisfaction. Model 3 showed that managerial roles not only affected job satisfaction, but that they could also have a moderating effect. Model 4 showed that managerial roles had a moderating effect on job satisfaction through interactions with job autonomy or job demand. The explanatory power of Model 4 increased by 0.1% compared to Model 3: Managerial roles had a negative moderating effect on the relationship between job autonomy and job satisfaction, thereby supporting H4. However, managerial roles had a positive moderating effect on the relationship between job demand and job satisfaction, thus also supporting H4.

Table 6 shows the results concerning the question of whether the effects of irregular employees’ job stress on job satisfaction were moderated by managers. The variable input for Models 1–4 was the same for both irregular and regular employees. The fit of all models for irregular employees was statistically significant. The explanatory power of Model 2 increased by 11.5% compared to Model 1: Job autonomy had a positive effect on job satisfaction, while job demand had a negative effect. The explanatory power of Model 3 increased by 15.2% compared to Model 2: Management had a positive effect on job satisfaction. The explanatory power of Model 4 increased by 0.4% compared to Model 3: Management had a negative moderating effect on the relationship between job autonomy and job satisfaction, and between job demand and job satisfaction, supporting H4.

## 5. Discussion

This study analyzed the characteristics of job stress and satisfaction, the effect of job stress on job satisfaction, and the effect of managerial roles on the same by employment type. The job autonomy of regular employees was found to be higher than that of irregular employees, and the job demand of irregular employees was found to be higher than that of regular employees, upon analyzing job autonomy and job demands (subfactors of job stress). The same results were also found when controlling for demographic characteristics, such as gender, age, academic background, and company size. Furthermore, job autonomy had a positive effect on job satisfaction, and job demand had a negative effect on job satisfaction. Thus, when taken together, these results showed that the job satisfaction of regular employees was higher than that of irregular employees.

For regular employees, the higher the educational background, the higher the job autonomy and lower the job demands. However, irregular employees did not have as consistent characteristics as regular employees. In the distribution of educational background, university graduates or higher accounted for the largest proportion of regular employees (63%), while high school graduates comprised the largest proportion of irregular employees (46.8%; χ^2^ = 4857.545, *p* < 0.001). For both regular and irregular employees, workload-control and decision-making (subfactors of job autonomy) had positive effects on job satisfaction, and time-pressure (a subfactor of job demand) had a negative effect on job satisfaction.

The majority of regular employees had university degrees or higher. A higher educational background has been shown to increase employees’ range of available job choices, the chances of getting a job in the company of their choosing, and chances of working in large companies [67,68,69]. It was reported that irregular employees mainly worked to support regular employees’ jobs, and even if they did independent jobs, they performed jobs that had goals to be accomplished on an hourly basis [70]. Therefore, job autonomy for irregular employees is inevitably limited, and job demand is inevitably high, as they mainly perform jobs whose wages are based on how many hours they work. These characteristics, related to the work environment, thus lead to differences in job autonomy and job demands between regular and irregular employees, and affect job satisfaction [20].

For both regular and irregular employees, the effect of job stress on job satisfaction was moderated by managerial roles, as managers could help to solve problems that might affect employee job performance [71,72,73]. However, upon analyzing job autonomy and job demand (subfactors of job stress), differences were found in the moderating effect of managerial roles on job satisfaction between regular and irregular employees. For the former, the interaction of job autonomy and managerial roles had a negative effect on the relationship between job autonomy and job satisfaction. In the relationship between job demand and job satisfaction, the interaction of job demand and managerial roles had a positive effect. As for irregular employees, the interaction of job autonomy and managerial roles, and of job demand and managerial roles, had a negative effect on the relationships between job satisfaction and both job autonomy and job demand.

Job autonomy can be defined as the freedom and discretion that employees have with respect to the goals, methods, and performance plans in performing the tasks that have been assigned to them [74]; this implies having the freedom and authority to make a wide range of decisions about procedures or methods required to complete jobs, and the order and timing of work, from the goal-setting stage [75,76]. In this study, as in many previous studies, job autonomy had a positive effect on job satisfaction. Therefore, managers intervening in employees’ job autonomy could be regarded as interference; hence, it could be said that managerial roles may have a negative effect on the interaction between job autonomy and job satisfaction in both regular and irregular employees.

Job demand means the degree of physical or psychological effort required by employees in performing their job [77], and generally includes factors such as time-pressure, excessive workload or poor working conditions [78]. Time-pressure was used as a variable of job demand in this study. As in previous studies, in this study, job demand was found to have a negative effect on job satisfaction. The present study showed that the interaction between job demand and managerial roles moderated the job satisfaction of employees. Among regular employees, the interaction between job demand and managerial roles had a positive effect on job satisfaction, while in irregular employees, the opposite was observed.

Regular employees tend to have loyalty toward their organization and are active in their job duties, due to job security. However, when the job is difficult to perform due to a high workload, or when high performance goals increase job stress, managers are expected to actively intervene and adjust job requirements in a reasonable and fair manner. In this situation, managers taking an active role to support regular employees who are under job stress due to job demands have a positive effect on job satisfaction by alleviating job stress [79]. However, the job demands placed upon irregular employees are generally determined from the time they start work. In such a situation, managerial intervention regarding job demands creates a new situation at work for an employee. Therefore, irregular employees view intervention from a manager as interference. Thus, managerial roles have a negative effect on the relationship between job demand and job satisfaction [80].

This study has some limitations. First, the subfactors that measure job stress can include variables such as inadequate rewards, job insecurity, and the physical workplace environment, in addition to job autonomy and job demand. However, this study was not able to conduct a broader analysis by including the relationship with job satisfaction using some subfactors (job autonomy and job demand) of job stress, as there was a limitation in collecting related data from the KWCS. Second, this study had a cross-sectional design. Based on a specific point in time, the mediating role of managers in the relationship between job stress and satisfaction was measured. However, the cross-sectional design makes it difficult to reflect factors that can change over time and according to a given situation. Third, this study classified regular and irregular employees based on data from the KWCS, and analyzed their characteristics and effects of managerial roles. However, if analyzed in more detail, such as by industry type and company size, the characteristics of regular and irregular employees and the effects of managerial roles may be different than what was found in the present study.

## 6. Conclusions

This study showed that there were differences in job stress and satisfaction by employment type, and that job stress had an effect on job satisfaction for both regular and irregular employees. Job stress and satisfaction affect not only individual employees, but also the performance of the organization; therefore, this must be addressed consistently. Managerial roles are very important with regard to employee job stress and satisfaction. However, this study showed that the moderating effect of the interaction between the factors of job stress and managerial roles differed by employment type in the relationship between job stress and job satisfaction. This implies that management should be applied differently according to employment type. To have a positive effect on job satisfaction for both regular and irregular employees, job autonomy should be guaranteed within the scope of the assigned job, and managerial intervention should be minimized. Nevertheless, in the case of regular employees, when they are under high levels of stress, proper intervention from a manager helps to relieve such stress and increase job satisfaction. However, in the case of irregular employees, the interaction between job demands and management has a negative impact on job satisfaction. Therefore, clear job descriptions should be provided when irregular employees begin work, and managerial intervention should be minimized. The results of the present study could be used as basic data for companies that are working to create policies to reduce job stress and increase job satisfaction for both their regular and irregular employees.

## Figures and Tables

**Table 1:** Reliability and validity of variables.

Variable	Item	Factor Analysis				Reliability
		Factor Loading	Communality	Eigenvalue	Explained Variance (%)	Cronbach’s α
Job autonomy	Workload-control	0.891	0.794	1.587	79.354	0.740
	Decision-making	0.891	0.794			
Job satisfaction	Wage	0.767	0.591	2.836	56.721	0.807
	Prospect	0.738	0.544			
	Recognition	0.816	0.667			
	Human relation	0.677	0.458			
	Motivation	0.760	0.577			
Manager role	Respect	0.772	0.597	3.754	62.570	0.880
	Compliment	0.790	0.624			
	Cooperation	0.793	0.629			
	Support	0.794	0.631			
	Feedback	0.793	0.628			
	Encouragement	0.804	0.646			

**Table 2:** Characteristics of job stress and satisfaction in regular and irregular employees.

Variable	Sub-Variable	Item	Regular Employees (*n* = 27,782)		Irregular Employees (*n* = 5638)		*t*	*p*
			Mean	SD	Mean	SD		
Job stress	Job autonomy		3.202	0.840	2.811	0.937	29.034	<0.001
		Workload-control	3.209	0.955	2.840	1.051	24.383	<0.001
		Decision-making	3.196	0.938	2.783	1.046	27.483	<0.001
	Job demand	Time-pressure	2.609	0.827	2.751	0.887	11.103	<0.001
Job satisfaction			3.507	0.535	3.204	0.603	35.062	<0.001

Note. SD: standard deviation, *t*: t-value.

**Table 3:** Job stress and satisfaction in regular and irregular employees according to demographic variables.

Variable	Characteristic	Classification	Regular Employees (*n* = 27,782)		Irregular Employees (*n* = 5638)		*t*	*p*
			Mean	SD	Mean	SD		
Job autonomy	Gender	Male	3.275	0.819	2.843	0.926	22.574	<0.001
		Female	3.091	0.859	2.784	0.947	16.948	<0.001
	Age	≤29 years	3.050	0.857	2.747	0.941	10.913	<0.001
		30–39 years	3.215	0.798	3.000	0.919	5.882	<0.001
		40–49 years	3.256	0.834	2.991	0.901	8.029	<0.001
		≥50 years	3.231	0.867	2.744	0.937	23.585	<0.001
	Academic background	Elementary school or lower	2.894	0.919	2.461	0.907	6.718	<0.001
		Middle school	2.968	0.935	2.704	0.919	5.788	<0.001
		High school	3.027	0.895	2.825	0.919	9.790	<0.001
		University or higher	3.286	0.797	2.980	0.948	12.351	<0.001
	Company size	≤49 employees	3.186	0.841	2.818	0.928	24.977	<0.001
		50–299	3.202	0.843	2.804	0.976	9.426	<0.001
		≥300	3.295	0.823	2.765	1.064	6.880	<0.001
Job demand	Gender	Male	2.586	0.815	2.776	0.883	10.389	<0.001
		Female	2.654	0.844	2.730	0.889	4.658	<0.001
	Age	≤29 years	2.652	0.834	2.753	0.870	3.901	<0.001
		30–39 years	2.605	0.801	2.701	0.899	2.699	0.007
		40–49 years	2.595	0.830	2.744	0.878	4.638	<0.001
		≥50 years	2.602	0.845	2.766	0.895	8.255	<0.001
	Academic background	Elementary school or lower	2.762	0.869	2.755	0.883	0.118	0.906
		Middle school	2.705	0.917	2.834	0.892	2.884	0.004
		High school	2.698	0.859	2.746	0.880	2.439	0.015
		University or higher	2.568	0.806	2.715	0.893	6.304	<0.001
	Company size	≤49 employees	2.626	0.832	2.743	0.885	8.256	<0.001
		50–299	2.590	0.820	2.788	0.873	5.213	<0.001
		≥300	2.541	0.800	2.868	0.969	4.652	<0.001
Job satisfaction	Gender	Male	3.522	0.535	3.161	0.602	28.971	<0.001
		Female	3.484	0.533	3.241	0.601	20.105	<0.001
	Age	≤29 years	3.467	0.527	3.228	0.597	13.666	<0.001
		30–39 years	3.544	0.532	3.271	0.611	11.296	<0.001
		40–49 years	3.531	0.525	3.276	0.588	11.911	<0.001
		≥50 years	3.465	0.547	3.152	0.604	23.661	<0.001
	Academic background	Elementary school or lower	3.228	0.530	3.119	0.574	2.748	0.006
		Middle school	3.207	0.578	3.035	0.618	5.841	<0.001
		High school	3.392	0.546	3.206	0.592	14.149	<0.001
		University or higher	3.569	0.515	3.322	0.601	15.787	<0.001
	Company size	≤49 employees	3.478	0.536	3.200	0.599	29.304	<0.001
		50–299	3.547	0.514	3.194	0.612	13.381	<0.001
		≥300	3.622	0.534	3.319	0.647	6.430	<0.001

**Table 4:** Effects of individual variables of job stress on job satisfaction on regular and irregular employees.

Category			Job Satisfaction (Regular Employees)			Job Satisfaction (Irregular Employees)		
			*β*	*t*	*p*	*β*	*t*	*p*
Control variable	Gender		0.001	0.242	0.809	0.067	5.390	<0.001
	Age		0.006	2.100	0.036	−0.013	−0.906	0.365
	Academic background		0.124	22.866	<0.001	0.079	5.285	<0.001
	Company size		−0.001	−2.127	0.033	0.019	1.520	0.129
Independent variable	Job autonomy	Workload-control	0.083	21.325	<0.001	0.176	11.237	<0.001
		Decision-making	0.054	13.799	<0.001	0.082	5.272	<0.001
	Job demand	Time-pressure	−0.117	−31.076	<0.001	−0.208	−16.323	<0.001
R^2^			139			142		
Adjusted R^2^			0.138			141		
F			559.165 (*p* < 0.001)			116.245 (*p* < 0.001)		

Note. *β*: Standardized Coefficient.

**Table 5:** The moderating effect of managerial roles in the relationship between job stress and satisfaction in regular employees.

Category		Job Satisfaction											
		Model 1			Model 2			Model 3			Model 4		
		β	t	*p*	β	t	*p*	β	t	*p*	β	t	*p*
Control Variable	Gender	−0.021	−3.572	<0.001	0.002	0.349	0.727	0.005	0.933	0.351	0.004	0.808	0.419
	Age	0.045	6.922	<0.001	0.013	2.184	0.029	0.018	3.275	0.001	0.020	3.503	<0.001
	Academic background	0.194	29.938	<0.001	0.141	22.585	<0.001	0.122	21.640	<0.001	0.122	21.639	<0.001
	Company size	−0.007	−1.176	0.239	−0.011	−1.937	0.053	−0.016	−3.180	0.001	−0.016	−3.168	0.002
Independent variable	Job autonomy (A)				0.211	35.284	<0.001	0.100	17.795	<0.001	0.302	8.657	<0.001
	Job demand (B)				−0.184	−31.212	<0.001	−0.092	−16.790	<0.001	−0.164	−4.885	<0.001
Moderating variable	Manager role (C)							0.428	77.100	<0.001	0.500	17.952	<0.001
Interaction	A*C										−0.259	−5.867	<0.001
	B*C										0.071	2.182	0.029
R^2^		0.040		0.137			0.292				0.293		
Adjusted R^2^		0.040		0.137			0.292				0.293		
⊿ R^2^		0.040		0.097			0.155				0.001		
F		226.639 (*p* < 0.001)		613.825 (*p* < 0.001)			1397.918 (*p* < 0.001)				1125.948 (*p* < 0.001)		

Note. *: it represents the interaction of two variables.

**Table 6:** The moderating effect of managerial roles in the relationship between job stress and satisfaction in irregular employees.

Category		Job Satisfaction											
		Model 1			Model 2			Model 3			Model 4		
		*β*	*t*	*p*	*β*	*t*	*p*	*β*	*t*	*p*	*β*	*t*	*p*
Control Variable	Gender	0.067	5.018	<0.001	0.070	5.568	<0.001	0.062	5.397	<0.001	0.062	5.457	<0.001
	Age	0.010	0.641	0.522	−0.012	−0.795	0.426	0.007	0.547	0.585	0.009	0.669	0.504
	Academic background	0.139	8.658	<0.001	0.087	5.725	<0.001	0.089	6.420	<0.001	0.093	6.703	<0.001
	Region	−0.035	−2.612	0.009	−0.033	−2.636	0.008	−0.037	−3.249	0.001	−0.038	−3.336	0.001
	Company size	0.022	1.618	0.106	0.025	1.954	0.052	0.036	3.129	0.002	0.035	3.043	0.002
Independent variable	Job autonomy (A)				0.225	17.067	<0.001	0.140	11.439	<0.001	0.508	6.882	<0.001
	Job demand (B)				−0.209	−16.071	<0.001	−0.103	−8.443	<0.001	−0.070	−1.089	0.276
Moderating variable	Manager role(C)							0.419	34.073	<0.001	0.669	12.717	<0.001
Interaction	A*C										−0.442	−5.054	<0.001
	B*C										−0.172	−2.730	0.006
R^2^		0.024			0.139			0.290			0.294		
Adjusted R^2^		0.023			0.137			0.289			0.293		
⊿ R^2^		0.024			0.115			0.152			0.004		
F		26.735 (*p* < 0.001)			124.678 (*p* < 0.001)			277.541 (*p* < 0.001)			225.888 (*p* < 0.001)		

Note. *: it represents the interaction of two variables.
